# [Retracted] MicroRNA‑217 functions as a tumor suppressor in cervical cancer cells through targeting Rho‑associated protein kinase 1

**DOI:** 10.3892/ol.2024.14657

**Published:** 2024-09-02

**Authors:** Jing Dong, Maoxiu Wang, Donghua Ni, Lixin Zhang, Wen Wang, Xiujuan Cui, Shijie Fu, Shujuan Yao

Oncol Lett 16: 5535–5542, 2018; DOI: 10.3892/ol.2018.9335

Following the publication of this paper, it was drawn to the Editor's attention by a concerned reader that certain of the western blotting data shown in Figs. 1A and B and 5A, the cell invasion assay data shown in Fig. 3B, and the flow cytometric assay data shown in Fig. 3C had already appeared in previously published articles written by different authors at different research institutes. Owing to the fact that the contentious data in the above article had already been published prior to its submission to *Oncology Letters*, the Editor has decided that this paper should be retracted from the Journal. The authors were asked for an explanation to account for these concerns, but the Editorial Office did not receive a reply. The Editor apologizes to the readership for any inconvenience caused.

